# Transcription Factor C/EBP Homologous Protein in Health and Diseases

**DOI:** 10.3389/fimmu.2017.01612

**Published:** 2017-11-27

**Authors:** Yuan Yang, Lian Liu, Ishan Naik, Zachary Braunstein, Jixin Zhong, Boxu Ren

**Affiliations:** ^1^Center for Molecular Medicine, Medical School of Yangtze University, Jingzhou, China; ^2^Department of Radiology, Medical School of Yangtze University, Jingzhou, China; ^3^Department of Pharmacology, Medical School of Yangtze University, Jingzhou, China; ^4^Cardiovascular Research Institute, Case Western Reserve University, Cleveland, OH, United States; ^5^Boonshoft School of Medicine, Wright State University, Dayton, OH, United States

**Keywords:** C/EBP homologous protein, apoptosis, endoplasmic reticulum stress, fibrosis, cancer, neurodegenerative disorders, diabetes

## Abstract

C/EBP homologous protein (CHOP), known also as DNA damage-inducible transcript 3 and as growth arrest and DNA damage-inducible protein 153 (GADD153), is induced in response to certain stressors. CHOP is universally acknowledged as a main conduit to endoplasmic reticulum stress-induced apoptosis. Ongoing research established the existence of CHOP-mediated apoptosis signaling networks, for which novel downstream targets are still being determined. However, there are studies that contradict this notion and assert that apoptosis is not the only mechanism by which CHOP plays in the development of pathologies. In this review, insights into the roles of CHOP in pathophysiology are summarized at the molecular and cellular levels. We further focus on the newest advances that implicate CHOP in human diseases including cancer, diabetes, neurodegenerative disorders, and notably, fibrosis.

## Introduction

C/EBP homologous protein (CHOP), also known as growth arrest and DNA damage-inducible protein 153 (GADD153), belongs to the CCAAT/enhancer-binding protein (C/EBP) family. Much of our understanding of CHOP originates from the roles it plays during endoplasmic reticulum (ER) stress (1) and amino acid limitation (2). It was gradually discovered as a stress-responsive transcription factor during growth arrest, DNA damage, nutrient deprivation, hypoxia, genotoxic agents, etc. CHOP expression is induced by unfolded protein response (UPR) and integrated stress response (ISR) (3, 4), primarily through the PRKR-like ER kinase (PERK) pathway. As a nuclear transcription regulator, CHOP also controls numerous genes involved in multifaceted cellular processes including inflammation, differentiation, autophagy, and apoptosis. A considerable aspect of CHOP’s involvement in disease is evident in the fact that sustained CHOP activation has long been accepted as a pivotal trigger for ER stress-related apoptosis.

In eukaryotic cells, the ER is a specialized organelle with the capacity for synthesis and storage of calcium as well as the folding and transport of secretory proteins to maintain cellular proteostasis. However, intrinsic and extrinsic insults, such as perturbations in calcium homeostasis and redox status, disturb ER proteostasis and cause accumulation of unfolded or misfolded proteins, collectively termed ER stress. In response, cells activate a series of adaptive pathways, namely the UPR, to restore homeostasis. Another innate protective pathway to proteostatic regulation is the ISR (5). Literature on the role of ER stress (or protein misfolding) and UPR in numerous disease states, such as cancer, neurodegenerative disease, metabolic disease, and genetic disorders, has been well reviewed (6–8). To penetrate into the significance of CHOP in pathological processes, it is important to have a full overview of several aspects of CHOP. In this review, we delineate its structure and characteristics. The regulating mechanisms of CHOP at the transcriptional level and its functions–primarily apoptosis are summarized in detail. Finally, the latest studies targeting CHOP will be highlighted in four classifications of human disease, with special attention to fibrosis, for which the targeting of CHOP as a therapeutic approach has not yet been reviewed.

## Characterization and Molecular Function of CHOP

**Figure 1:**
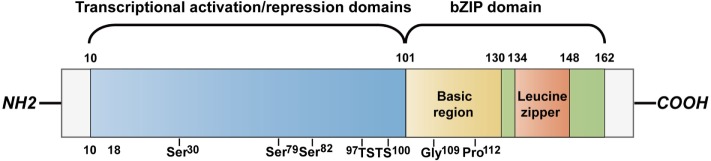
CHOP structure. CHOP is a protein containing 169 amino acids that divide into N-terminus transcriptional activation/repression domains and a C-terminus bZIP domain including a basic region for DNA binding and a leucine zipper region for dimerization. The motif between aa 10 and 18 is for interaction with TRB3. The transactivation domain contains a serine residue (30) that is phosphorylated by AMPKα1 to trigger the proteasomal degradation of CHOP in macrophages. It is also degraded by SPOP that recognizes the serine/threonine-rich motif between aa 97 and 100. Phosphorylation at two serine residues (79, 82) by p38 MAPK enhances the transcriptional activation by CHOP. The basic region holds glycine (109) and proline (112) substitutions interrupting the DNA-binding activity. CHOP, C/EBP homologous protein; bZIP, basic-leucine zipper; SPOP, speckle-type POZ protein; p38 MAPK, p38 mitogen-activation protein kinase; aa, amino acid.

C/EBP homologous protein, encoded by the DNA damage-inducible transcript 3 (*Ddit3*) gene, is one of the six identified members of C/EBP trans-acting factors that bind to the CCAAT box motif present in several promoters. CHOP is characterized by transcriptional activation/repression domains at its N-terminus and a C-terminus basic-leucine zipper (bZIP) domain which contains a basic region mediating sequence-specific DNA binding along with a leucine zipper motif for dimerization. The N-terminal region is necessary for proteasomal degradation of CHOP. A serine/threonine-rich motif (97–100) in its transactivation domain can be recognized by speckle-type POZ protein (SPOP), which triggers CHOP degradation *via* the ubiquitin–proteasome pathway (9). Similarly, macrophage AMP-activated protein kinase α1 mediates CHOP ubiquitination and proteasomal degradation *via* phosphorylation at the serine residue (30) (10). Two serine residues (79, 82) are responsible for CHOP phosphorylation by p38 mitogen-activation protein kinase (p38 MAPK) (11). This phosphorylation event enhances its transactivation activity and is required for CHOP-induced apoptosis in macrophages (12) (Figure 1). It is well known that the conservation of CHOP’s bZIP domain provides a platform for the formation of heterodimers. Furthermore, the basic region of CHOP holds proline and glycine residues that interrupt DNA-binding activity of the protein, causing increased heterodimerization with other C/EBPs (13). The homotypic heterodimers uniquely bind to the sequence (A/G)TGCAAT(A/C)CCC in response to stress (14). CHOP can also dimerize with members of another bZIP subgroup, the CREB/activating transcription factor (ATF) family. Consistently, a C/EBP-ATF-binding site is present in the amino acid response elements (AARE) of *CHOP* promoter (15). It has also been reported that the bZIP domain is required for CHOP-induced apoptotic processes (16, 17). Tribbles-related protein 3 (TRB3) recognizes the region between amino acid (aa) 10 and 18 to interact with CHOP.

C/EBP homologous protein serves as a double-edged transcription factor. It was originally proposed to be a dominant-negative regulator for other C/EBP-induced transcription by forming dimers and impairing their DNA-binding activity (18). However, CHOP also negatively regulates ATF4-dependent induction of the *ASNS* gene during ER stress or amino acid deprivation (19). Indeed, microarray analysis shows CHOP overexpression inactivates the expression of most of the target genes, serving as a dominant-negative factor by sequestration of dimer forming transcription factor partners (20). Nevertheless, subsequent studies have shed light on the positive role of CHOP–C/EBP interaction in transcriptional activation (21, 22) and have also revealed that CHOP–ATF4 heterodimers induce the expression of numerous stress-responsive genes (23).

## Regulation of CHOP

**Figure 2:**
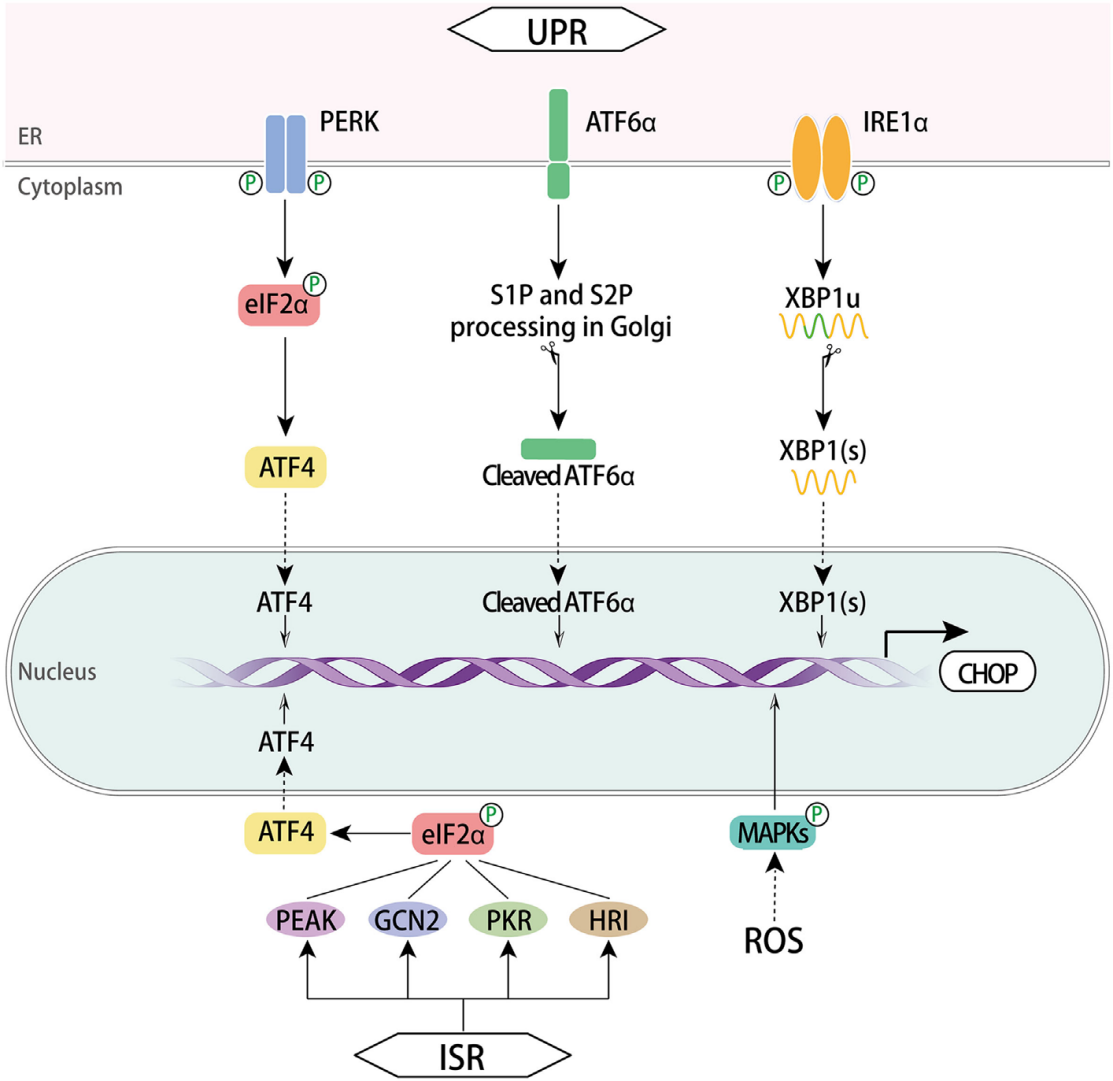
Regulation of CHOP. The three signaling branches of UPR lead to CHOP transcription respectively. Once activated *via* dimerization and trans-autophosphorylation, PERK phosphorylates eIF2α, which enables ATF4 translation. Subsequently, CHOP is activated by ATF4 trafficking to the nucleus. In the presence of misfolded proteins, ATF6α translocates to the Golgi apparatus where it was processed by the protease SP1 and SP2, thus producing a cytosolic fragment ATF6f to regulate CHOP activation in the nucleus. Activation of IRE1α RNase domain processes unspliced XBP1 mRNA to create activated XBP1(s), which enters the nucleus and controls the expression of CHOP. Another pathway involves ISR. This response is initiated with GCN2, PKR, HRI, and PEAK that converge on the phospho-eIF2α/ATF4 pathway and CHOP induction ensues. A ROS-dependent mechanism also activates CHOP *via* MAPKs. CHOP, C/EBP homologous protein; UPR, unfolded protein response; ISR, integrated stress response; ATF, activating transcription factor; ATF6α, activating transcription factor 6α; PERK, PRKR-like ER kinase; XBP1, X box-binding protein 1; GCN2, general control nonderepressible 2; PKR, RNA-dependent protein kinase; HRI, heme regulated inhibitor; ROS, reactive oxygen species; MAPKs, mitogen-activated protein kinases; eIF2α, eukaryotic translation initiator factor 2α.

C/EBP homologous protein is a cellular stress sensor that can be induced in response to a series of physiological or stress conditions such as ER stress, nutrient deprivation, DNA damage, cellular growth arrest, and hypoxia (1, 2, 24). It expresses at a very low level in normal physiology, but cellular stress leads to high-level expression. CHOP is acknowledged as a specific and convergent transcription factor of ER stress and its expression is generally modulated at the transcriptional level. CHOP transcription can be regulated *via* ER stress response elements (ERSE) and the C/EBP-ATF response element (CARE) of its promoter in response to cellular stress (15, 25), and *via* amino acid response elements (AARE) under amino acid starvation conditions (26) (Figure 2).

### Unfolded Protein Response

Endoplasmic reticulum stress induces UPR, an adaptive mechanism that controls cell fate between survival and death in an intensity time-dependent manner. It involves three signal transduction pathways initiated by three ER transmembrane proteins: PERK, inositol requiring protein 1α (IRE1α, also known as ERN1), and activating transcription factor 6α (ATF6α) (27, 28). Each of the three maintains an inactive state in combination with the ER chaperone BiP (also named GRP78) in resting cells. Under chronic or overwhelming ER stress, all three mammalian UPR pathways uniquely lead to the initiation of CHOP transcription through binding sites within *CHOP’s* promoter. The cis-acting AARE1 and AARE2 as well as the composite CARE (29) are bound by ATF4, while ATF6α and X box-binding protein 1 (XBP1) bind to ERSE1 and ERSE2.

The PERK pathway is predominant in CHOP activation. Upon the luminal binding of misfolded proteins, PERK is activated through dimerization and trans-autophosphorylation (30). It phosphorylates eukaryotic translation initiator factor 2α (eIF2α), which then attenuates global protein synthesis. Upon activation, ATF4 translocates into the nucleus and transcriptionally upregulates *CHOP* as well as many UPR genes that are vital for amino acid metabolism and redox processes (31).

The activation of IRE1α is similar to PERK in that its luminal domains are first dimerized and then are trans-autophosphorylated. Activated IRE1α creates spliced XBP1(s) by cleaving a 26-nucleotide intron from the mRNA of unspliced XBP1 using a cytoplasmic RNase domain. IRE1α also mediates regulated IRE1-dependent decay of selective mRNAs (32). XBP1(s) enters the nucleus and induces the transcription of genes correlated with protein-folding capacity and ER-associated degradation. Hence, CHOP expression is upregulated by XBP1(s) (33, 34).

In the ATF6α branch, the type II ER located protein ATF6α is transported to the Golgi apparatus where it is processed by Site-1 and Site-2 proteases (SP1 and SP2, respectively). As a consequence of this processing, a cytosolic fragment of ATF6α is produced and enters the nucleus to regulate the expression of target genes, including *BiP* and *CHOP* (35, 36). Along with XBP1(s), ATF6f contributes to the augmentation of ER size and ER protein-folding capacity through target genes.

### Integrated Stress Response

Integrated stress response serves as another cytoprotective mechanism against various stressors, such as ER stress, nutrition stress, oxidative stress, proteasome inhibition, hyperoxia, or viral infection (5, 37, 38). This common adaptive response initiates with four kinases consisting of general control nonderepressible 2, RNA-dependent protein kinase (39), heme regulated inhibitor, and PEAK, all four of which then converge on a core event, the phospho-eIF2α/ATF4 pathway, which in turn increases the transcription of CHOP (4, 40).

### Reactive Oxygen Species (ROS)–MAPKs

Reactive oxygen species disturb redox status and ER homeostasis, thus inducing ER stress responses. ROS have been reported to activate CHOP through the AP-1 element in the CHOP promoter (41). The MAPKs consisting of JNK, p38 MAPK, and ERK are canonical downstream mediators of ROS (42). There are a number of studies establishing the signaling axis of ROS-induced CHOP upregulation *via* MAPKs signaling pathways in different cells. The ROS–MAPKs–CHOP pathway has been reported to suppress migration of hepatocellular carcinoma (HCC) cells (43) and mediate the downstream death receptor pathway in a number of cancer cells (44–47). Moreover, a scavenger of ROS diminished the PERK/eIF2α/CHOP pathway (48). IRE1 can recruit TRAF2 to activate ASK1, which can, through different pathways, induce expression of JNK and p38 MAPK (49). Phosphorylation of CHOP by p38 MAPK is required for its activation. Thus, the way by which ROS activates MAPKs and CHOP may be the IRE1α or PERK pathway of UPR (50). The ATF4/ATF3 axis was also reported to induce CHOP expression under ROS-dependent ER stress (51, 52).

### Others

Several members of the CREB/ATF transcription factor family are capable of regulating CHOP expression. ATF3 can interact with the CARE elements within the *CHOP* promoter (29), while ATF5 activates the AARE1 site (53) under arsenite exposure. ATF2 binds the AARE sequence to regulate *CHOP* transcription in response to amino acid starvation (54). Conversely, some factors can inhibit the expression of CHOP, thereby reducing its detrimental effects. At early stage of ER stress, miR-211 expression, induced by PERK activation, can suppress *CHOP* transcription through histone methylation at its promoter (55). CHOP expression can also be repressed through toll-like receptor (TLR)–TRIF-dependent pathway which increases the activity of eIF2B to counteract the effect of p-eIF2α under the treatment of LPS, a TLR4 ligand *in vitro* and *in vivo*. Furthermore, the activation of eIF2B by TLR–TRIF signaling is attributed to serine dephosphorylation of eIF2Bε by protein phosphatase 2A. When TRIF was deficient in mice, CHOP induction, apoptosis, and organ dysfunction ensued (56, 57).

## Cellular Function of CHOP

In addition to its pro-apoptotic role, the function of CHOP in regulating other cellular processes has recently come to light. CHOP serves as a multifunctional transcription factor that contributes to cellular functions including apoptosis, autophagy, inflammation, cell differentiation, and proliferation. Under non-stressed conditions, the subcellular location of CHOP is mainly in the cytoplasm where it negatively affects cell migration-associated genes, while stress conditions lead to its nucleus translocation, partly *via* LIP, a C/EBPβ isoform (58), and its DNA-binding capacity therefore allows it to regulate gene expression. Nuclear CHOP can induce a transient cell cycle arrest in G_1_ phase (20). During ER stress, CHOP also inhibits the growth arrest-specific p20K genes, which are a group of genes that activate reversible G_0_ arrest to regulate cell proliferation (59). Generally, it is known as an important node in the transcription factor network that dominates stress-inducible regulation of specific target genes. *CHOP* deficiency does not produce a substantial phenotype without a stress signal.

### CHOP in Apoptosis Modulation and Signaling

**Figure 3:**
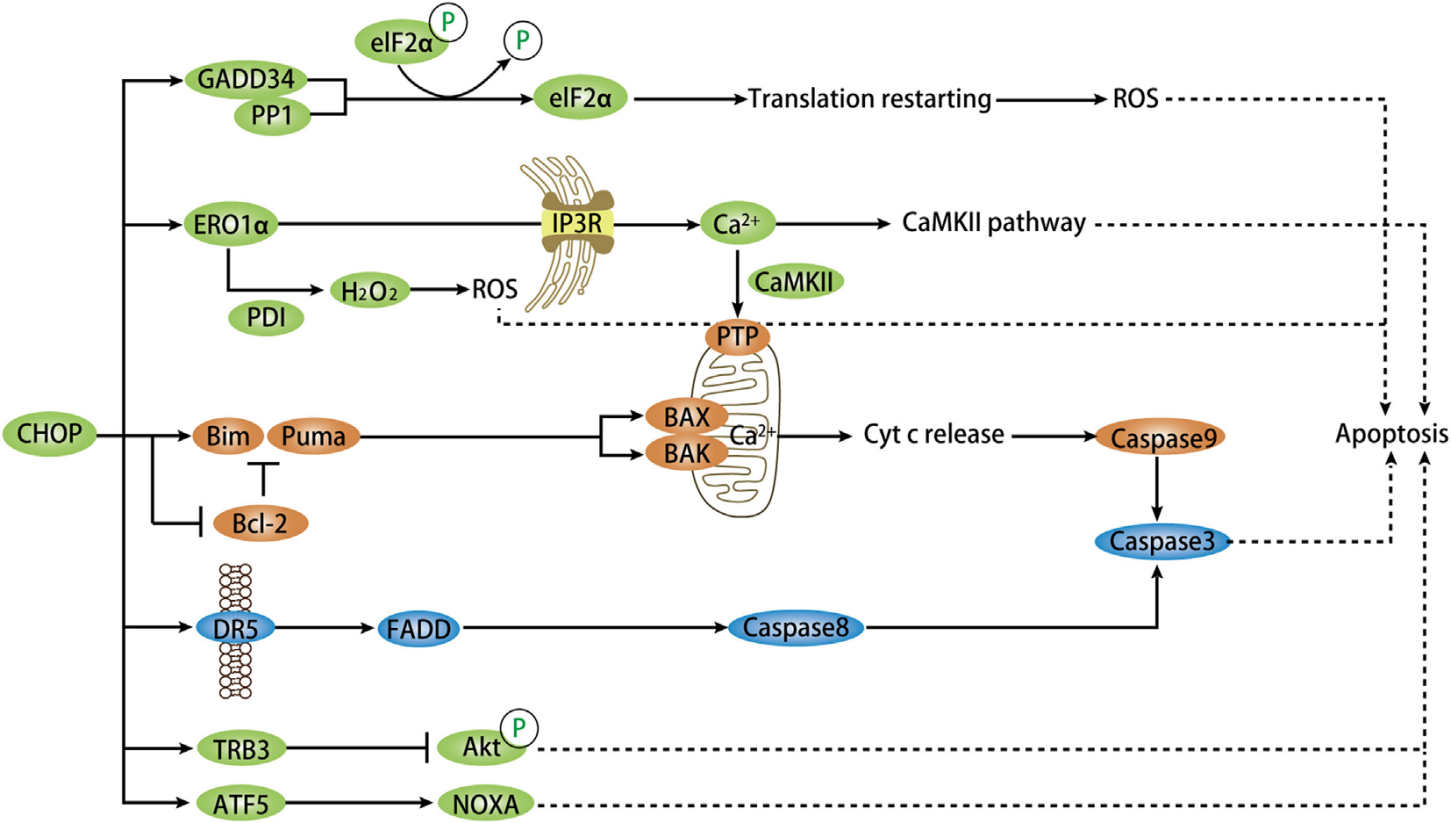
Model depicting targets of CHOP-dependent apoptosis. During chronic ER stress, CHOP activation mediates pro-apoptosis signaling *via* numerous targets and pathways directly or indirectly. CHOP triggers the intrinsic apoptotic pathway through inhibition of BCL-2 and upregulation of BIM and PUMA, which regulate BAX–BAK-mediated mitochondrial outer membrane permeabilization. This leads to cytochrome *c* release and caspase cascade. CHOP also directly induces the expression of DR5-mediating extrinsic apoptotic pathway *via* FADD and caspase8-mediated cascade. In normal conditions, CHOP-dependent ERO1α induction oxidizes PDI to produce ROS that plays a critical role in apoptosis. The ERO1α–IP3R–Ca^2+^–CaMKII pathway, in addition to ROS, can trigger several apoptotic pathways, primarily the Ca^2+^-dependent mitochondrial apoptosis *via* PTP. GADD34 is a key target of CHOP and ATF4 and combines with PP1 to promote dephosphorylation of phospho-eIF2α. This event renews protein translation that promotes apoptosis in certain stress settings. Another target is TRB3 that prevent Akt phosphorylation in this apoptotic pathway. ATF5, downstream of CHOP, facilitates apoptosis through activation of some pro-apoptotic genes, such as NOXA. CHOP, C/EBP homologous protein; ER, endoplasmic reticulum; ATF, activating transcription factor; ROS, reactive oxygen species; DR5, death receptor 5; FADD, Fas-associated death domain; GADD34, growth arrest and DNA-damage-inducible protein 34; PP1, protein phosphatase 1; PDI, protein disulfide isomerase; CaMKII, Ca^2+^/calmodulin-dependent protein kinase II; TRB3, tribbles-related protein 3; ERO1α, ER oxidase 1α.

During a stress situation, UPR attempts to increase protein-folding capacity and remove misfolded and unfolded proteins. If the remedy is inadequate to restore homeostasis under chronic ER stress, terminal UPR will trigger apoptosis through abundant signaling mechanisms, mainly mediated by CHOP, JNK, and caspase-12, with CHOP as the most widely studied (60). It is notable that CHOP expression itself is not sufficient to induce apoptosis unless exposed to a stress signal. Studies in both cellular and animal models with *CHOP* gene deficiency have shed light on the pro-apoptotic role of CHOP during cellular stress (61, 62). Synoptically, CHOP-dependent apoptosis is mainly mediated by altering the expression of pro-apoptotic or anti-apoptotic genes, either directly or indirectly (1, 63). Both the intrinsic, mitochondrial pathway and extrinsic, death receptor pathway of classic apoptosis can be activated by CHOP and proceed with a set of initiator caspases and common executioner caspases (64). Overall, the apoptotic pathways mediated by downstream targets of CHOP form networks (Figure 3), wherein the molecular interaction mechanisms remain to be further understood.

#### Bcl-2 Family

The B-cell lymphoma 2 (BCL2) family of proteins includes anti-apoptotic members, such as BCL2-like, and pro-apoptotic members, such as BH3-only and BAX-like. As a widely cited mechanism for the mitochondrial apoptotic pathway, CHOP induces the upregulation of certain BH3-only proteins, such as BIM (65), PUMA (66), while inhibiting the expression of BCL2 to release its sequestration of BH3-only proteins (67). Thus, they regulate BAX–BAK homo-dimerization and consequent mitochondrial outer membrane permeabilization, causing release of cytochrome *c* and stimulation of an apoptotic signaling cascade (68). A recent study pointed to a role for BOK, another BAX-like protein, in regulating ER stress-induced apoptosis through CHOP as evidenced by diminished activation of CHOP, diminished activation of BIM and apoptosis in Bok^−/−^ mice (69).

#### Death Receptor 5 (DR5)

C/EBP homologous protein has been proven to directly control the transcription of the TNF family member cell-surface DR5, which activates the adaptor Fas-associated death domain to trigger caspase8-induced apoptosis (70). The CHOP–DR5 model sensitizes several chemically challenged cancer cells to extrinsic apoptosis mediated by ROS *in vitro* (44, 71), and ATF4 both *in vivo* and *in vitro* (72). During this process, the upstream signaling of CHOP includes ATF4, ROS–MAPKs, or the ATF4–ATF3 axis (46, 52).

#### Growth Arrest and DNA-Damage-Inducible Protein 34 (GADD34)

During prolonged ER stress, PERK-induced CHOP expression directly upregulates the transcription of GADD34, which forms a complex with its cofactor protein phosphatase 1 to facilitate dephosphorylation of phospho-eIF2α and protein translation (63, 73). However, if the feedback protein synthesis increase does not revert proteostasis, GADD34 upregulation can cause further misfolded proteins aggregation and ROS production to promote apoptosis.

#### ER Oxidase 1α (ERO1α)

Reactive oxygen species-dependent oxidative stress in ER stress is induced by the CHOP target ERO1α (74). Normally, ERO1α is responsible for disulfide bond formation by oxidizing protein disulfide isomerase. This process is coupled with the production of hydrogen peroxide (H_2_O_2_), which raises ROS generation and causes cell death if excessively produced (75). This is in line with another report on CHOP knockdown that showed decreased H_2_O_2_ formation and ROS-induced apoptosis (76). Faced with stress, CHOP activates calcium-mediated apoptosis through ERO1α, which activates inositol-1,4,5-trisphosphate receptor (IP_3_R), the ER calcium channel that mediates Ca^2+^ efflux (77). As a result, the cytosolic Ca^2+^ activates Ca^2+^/calmodulin-dependent protein kinase II (CaMKII) that triggers multiple apoptotic pathways including JNK signaling. The ROS signal generated from one of these pathways can conversely amplify CHOP activation *via* a positive feedback loop (31). Another pathway is that of CaMKII, which promotes the uptake of Ca^2+^ by mitochondria through mitochondrial permeability transition pores to activate a mitochondrial apoptotic pathway (78). Collectively, ERO1α induces oxidative stress and Ca^2+^-mediated mitochondrial impairment in ER-stressed cells, which lead to CHOP-dependent apoptosis.

#### Tribbles-Related Protein 3 (TRB3)

Past research has demonstrated that CHOP–ATF4 cooperates to transactivate the transcription activity of TRB3 that contributes to CHOP-induced apoptosis in various cells types, such as cardiomyocytes (79). TRB3 binding to prevent Akt phosphorylation is probably the underlying mechanism of its pro-apoptotic function (80). The binding site of CHOP overlaps the amino-acid response elements in TRB3 promoter and respective specific regions in CHOP and TRB3 protein are responsible for their interaction (81).

Han and his colleagues have proposed a novel mode of CHOP-induced apoptosis where CHOP–ATF4 heterodimer binds to promoters of genes involved in protein synthesis, such as Gadd34, Trb3, Atf3, and Wars. Forced expression of CHOP and ATF4 evokes increased protein synthesis, consequent ATP depletion, and oxidative stress, thereby leading to cell death (23). This is in line with previous research on CHOP knockouts that showed less protein aggregation, ROS, and apoptosis (63, 82). Advances made in understanding the mechanism of ER stress-induced apoptosis have recently gained new impetus for the analysis of microRNAs in cells challenged with the ER stressor tunicamycin (TUN). For example, the miR-216b is proved to be a direct target of CHOP and thereby executes its pro-apoptotic activity by suppression of c-Jun expression (83). Full induction of ATF5 expression requires the upstream regulation of CHOP and the CARE element of its promoter can be bound by ATF4 and CHOP. As such, ATF5 potentiates CHOP-dependent apoptosis through activation of pro-apoptotic genes, including NOXA, during proteostasis imbalance (84). Another novel target of CHOP-mediated apoptosis is p21, a prominent cell cycle regulator with strong anti-apoptotic activity. The direct suppression of p21 transcription by CHOP is important for the pro-apoptotic pathway *in vivo* and *in vitro* (85, 86). CHOP also binds to the promoter of lipocalin 2, which mediates apoptosis in lung cancer cells in response to ER stress (87).

Recent research has also shown that CHOP does not elicit apoptotic processes under stress conditions in some specific cell types, such as myeloid-derived suppressor cells (MDSCs) (88) and myelinating glial cells (89). In light of the data, a distinct model of apoptosis proposed with an UPR cycle places CHOP in an obligatory step upstream of GADD34 that dephosphorylates p-eIF2α and resumes global protein synthesis, which is the decisive matter of cell fate (89). In this context, cells attempt to restore homeostasis while events including calcium loss, ATP depletion, and oxidative stress, eventually lead to cell death. If cells survive these events, protein aggregation due to subsequent stress drives another UPR cycle. This is consistent with a previous study in mouse embryonic fibroblasts (MEFs) overexpressing CHOP that implies forced expression of CHOP alone is not sufficient to induce apoptosis (23).

### Autophagy

To cope with an inadequate protein-folding environment in ER, cells activate autophagy, an early stress-adaptive self-eating process, responsible for lysosome-dependent degradation of protein aggregates and other cellular material through UPR signaling (90). Many cellular stresses can trigger autophagy or apoptosis depending on specific circumstances and autophagy usually precedes apoptosis. CHOP has been implicated in autophagy induced by amino acid starvation, ER stress, virus infection, and hypoxia.

During amino acid starvation and ER stress, CHOP binds to the promoters of a set of autophagy genes (91). The time course analysis provides a further understanding that its upregulation of autophagy genes is within a short period of leucine starvation in cells. However, as time goes on, CHOP turns to inhibit the activation of these genes. It also inhibits autophagic flux and the conversion of microtubule-associated protein 1A/1B-light chain 3B (referred to as LC3), which is a key step for autophagy formation. The UPR-controlled balance of cell fate is therefore inclined toward cell death (92). Moreover, CHOP modulates the induction of autophagosomes during ER stress, as evidenced by the inhibition of LC3-II expression and GFP-LC3B dots (93). The results also showed that CHOP upregulated IRE1α, which contributed to autophagy induction, but their specific role and relation in this autophagy pathway remain unknown. Similarly, CHOP-mediated Licochalcone A-induced autophagy in non-small cell lung cancer cells and HeLa cells, while knockdown of CHOP reversed autophagy by reducing LC3-II and GFP-LC3 expression (94). Another study implied that UPR-activated CHOP elicited complete autolysosome maturation in hepatitis C virus-induced autophagy *via* LC3B-II-dependent mechanism (95). In the context of hypoxia, CHOP induces the expression of the autophagy gene *Atg5* by directly binding to its promoters (96).

### Cell Differentiation

C/EBP homologous protein is involved in the block of differentiation in mesenchymal lineages. It is a fundamental regulator of adipogenesis, a role that has been supported by numerous experiments. The terminal differentiation of adipocytes is necessary for efficient lipid storage. CHOP was initially found to negatively regulate adipocyte differentiation *in vitro*, in response to metabolic stress (97), hypoxia (98), and when phosphorylated by stress-induced p38 MAPK (11). In subsequent studies, both *in vivo* and *in vitro* adipocyte differentiation is inhibited by CHOP expression after PERK–eIF2α activation during ER stress (99). As for the specific mechanisms, under polyamine depletion, CHOP exerts the inhibitory effect through interaction with C/EBPβ, thus impairing its role in the execution of mitotic clonal expansion process and in the transcriptional activation of peroxisome proliferator-activated receptor γ (PPAR-γ) and C/EBPα; the predominant regulators of adipogenesis (100). This is consistent with the molecular mechanism whereby CHOP impairs the differentiation of preadipocyte in response to aging (101). Higher levels of CHOP have led to hyperplasia of adipose tissue with less differentiated adipocytes in mouse models and downregulation of CHOP mRNA is required for complete adipocyte differentiation of MEFs (102).

Besides adipocytes, CHOP was also identified as a negative modulator for osteoblast differentiation. In terms of the mechanism, CHOP inhibits the binding activity of C/EBPβ against Runx2, thus suppressing their induction of the osteocalcin (*Ocn*) gene and phosphatase activity (103). This negative modulation is also supported by recent experiments. Osteoblast proliferation and differentiation can be regulated by ADP-ribosylation-like factor 6 interacting protein 5 (Arl6ip5), whereas CHOP is required for reduction of the above two events and induces apoptosis in Arl6ip5-knockdown osteoblasts (104). The promotion of osteoblast differentiation by the transcription factor EB is coupled with reduced expression of ATF4 and CHOP. However, their expression was upregulated by TFEB overexpression in the stimulation of bone morphogenic protein 2 (BMP2), a potent inducer of osteoblast differentiation (105). It was previously proposed that CHOP may indirectly promote BMP2-induced osteogenesis (103) and enhance osteoblast differentiation of mesenchymal progenitor cells especially in the presence of BMP2 (106). Consistent with the finding, the PERK–eIF2α–ATF4 pathway promotes BMP2-induced osteoblast differentiation (107). Taken together, expression of CHOP exerts a dual role in osteoblast differentiation. It may be that the inhibitory action of CHOP is not sufficient to affect BMP2-induced differentiation processes. The specific association between CHOP and BMP2 in osteoblasts remains a topic of further exploration. Moreover, myoblast differentiation is inhibited by CHOP, which suppresses the transcription of myogenic regulatory factor in myoblasts by binding to its transcription regulatory sequences and affecting histone acetylation (108). Recent evidence has also unveiled that CHOP blocks the progression of myeloid lineage in granulo-monocytic progenitors (109).

## CHOP in Diseases

C/EBP homologous protein has widely documented roles in metabolism, neurodegeneration, and therioma. Recent findings have added the condition of fibrosis to this list of diseases mediated by CHOP. CHOP-mediated cellular apoptosis leads to organ dysfunction and may thus be involved in a wide range of diseases. We herein highlight recent advances that implicate CHOP in the occurrence, development, and outcome of diseases; in addition to outlining potential treatment strategies that target CHOP.

### Fibrosis

Fibrosis progresses as an eventual pathological outcome of three stages of general wound-healing responses; injury, inflammation, and repair to persistent organ injury. A pathological hallmark of fibrosis is the excessive deposition of extracellular matrix (ECM) in the tissues. This increased protein synthesis may disturb ER homeostasis and induce the expression of CHOP (110). An emerging role of CHOP in promoting fibrotic response of internal organs is supported by alleviation of fibrosis in *CHOP*-knockout mice (111). Given that apoptosis is a common cellular event that leads to organ remodeling and fibrosis after insult, CHOP-initiated pro-apoptotic activity may be partly the underlying mechanism. Moreover, alternatively activated phenotype (M2) macrophages are considered to participate in promoting collagen deposition and fibrogenesis. In recent years, research advances have indicated that CHOP likely regulates the activation of M2 macrophages to trigger tissue fibrosis (112). These M2 macrophages would release various cytokines to create a microenvironment that favors fibrogenesis, including high levels of TGF-β1, which is a key factor that promotes the activation of ECM-producing myofibroblasts (113). Modulation of CHOP expression may be a potential treatment for organ fibrosis. Here, we show the vital role of CHOP in the formation of specific fibrotic disorders.

#### Lung Fibrosis

The crucial events in the pathogenesis of lung fibrosis include TGF-β activation and alveolar epithelial cells (AECs) apoptosis, which then trigger resident fibroblast proliferation and differentiation of ECM-producing myofibroblasts (114). Research in the past decade has established a relation between ER stress and lung fibrosis (115). CHOP, the key player in ER stress, was found to mediate methamphetamine (MA) and thrombin-induced apoptosis of AECs in chronic pulmonary injury or fibrotic lung tissue (116, 117). Likewise, analysis of lung tissue from mouse fibrosis models induced by bleomycin (BLM) and from patients with IPF has manifested altered CHOP expression along with ER stress. Indeed, the loss of CHOP expression protected mice from BLM-induced pulmonary injury and fibrosis (112, 118). Mechanistic investigation indicates that CHOP regulates the production of M2 macrophages and subsequent TGF-β1 signaling involved in lung fibrosis. Further studies dissected the mechanism through which *CHOP* deficiency reduced M2 macrophage infiltration, for it upregulated the STAT6 inhibitors SOCS1 and SOCS3, thus repressing STAT6/PPAR-γ signaling (112). Meanwhile, a different viewpoint has been posited that CHOP-mediated macrophage apoptosis provides protection for *Grp78*^+/−^ mice against BLM-induced fibrosis (119). Nevertheless, both of these competitive findings support the role of CHOP in regulating macrophage to participate in the progress of lung fibrosis.

#### Kidney Fibrosis

Renal fibrosis, which includes glomerulosclerosis and/or tubulointerstitial fibrosis, is a common pathogenic consequence of chronic progressive renal diseases. Overwhelming expression of ER stress markers including CHOP are associated with fibrosis in rat kidneys subjected to unilateral ureteral obstruction (UUO) (120). Zhang and colleagues first noted that *CHOP^−/−^* mice were protected from UUO-induced renal fibrosis, wherein loss of CHOP decreased UUO-induced apoptosis of tubular cells and the Hmgb1/TLR4/NFκB/IL-1β signaling. Thus, the IL-1β downstream TGF-β1/Smad2/3 signaling was also inhibited, eventually ameliorating renal fibrosis (121). Consistent results were also obtained that indicate that *CHOP* deletion attenuated renal tubulointerstital fibrosis in the mouse UUO model. In addition, there are novel findings about these mechanisms that *CHOP* deficiency not only lessens tubular cell apoptosis but also abates profibrotic factors, oxidative stress, and recruitment of inflammatory cells including macrophages (111). Recently, both in patients with renal fibrosis resulting from chronic kidney disease (CKD) and a mouse fibrosis model of hypertensive CKD, an increase in *CHOP* gene was demonstrated. Furthermore, inhibition of CHOP by an ER stress inhibitor, 4-phenylbutyric acid, attenuated renal interstitial fibrosis, as well as macrophage infiltration and TGFβ1 expression. *CHOP*-knockout mice developed less renal fibrosis accompanied by lower macrophage infiltration (122). Moreover, Pan et al. found that M2 macrophages specifically enhance epithelial-to-mesenchymal transition and subsequent renal fibrosis by high production of TGFβ1 in a mouse UUO model (113). In addition, a function of CHOP in inducing fibronectin production in tubule cells was identified, indicating its role in promoting tubulointerstitial fibrosis during diabetic nephropathy (DN) (123).

#### Liver Fibrosis

Hepatic stellate cells (HSCs) are the principal cell-type responsible for ECM production and collagen deposition during liver fibrogenesis in which it is activated into myofibroblast in a TGF-β1-dependent manner. An early study on hepatic fibrosis in cholestatic liver injury caused by bile duct ligation showed that *CHOP* deletion alleviated hepatocyte death and hepatic fibrosis, with inhibitory effect on TGF-β1 induction and HSCs activation (124). *CHOP* deficiency also attenuated liver fibrosis in HCC induced by diethylnitrosamine (DEN) (125), and a fat-loading, methionine-choline-deficient diet (126). As illustrated by the aforementioned studies, hepatocyte apoptosis is a cellular mechanism underlying the promotion of CHOP for hepatic fibrotic response (127). Moreover, CHOP was significantly upregulated in the liver from animals with CCl_4_-induced fibrosis (128). Another study showed that pronounced CHOP expression was stimulated by hepatitis B virus (HBV) surface proteins and correlated with increased liver injury and fibrosis in HBV transgenic mice on BALB/c background, as compared to C57BL/6 (129). Ablation of *CHOP* attenuated hepcidin suppression and ensuing iron overload in a mouse liver fibrosis model induced by thioacetamide (130). In contrast, liver fibrosis induced by dietary steatohepatitis was greater in *CHOP^−/−^* mice, due to lessened CHOP-induced apoptosis of activated macrophages (131).

Taken together, in both mouse renal fibrosis and pulmonary fibrosis models, the deletion of the *CHOP* gene resulted in a marked decrease in inflammatory infiltration of macrophages, embodying a reduced differentiation of M2 macrophages, which are considered as a new cell-type involved in fibrogenesis. To the best of our knowledge, no studies have yet found that CHOP can regulate M2 macrophages in hepatic fibrosis, while it has been verified that M2 macrophages play an important role in schistosomiasis-induced liver fibrogenesis *via* IL-13/STAT6 signaling pathways (132). Hepatic fibrosis during schistosomiasis may represent a class of special cases where macrophage is actively involved in host immune responses against schistosome infection. Macrophages have long been accepted as profibrotic in schistosome infection. However, there are reports showing that restorative macrophages, characterized by an anti-inflammatory anti-fibrogenic expression profile, are in fact key to the remodeling and resolution of liver fibrosis (133, 134). Therefore, further studies investigating the role of CHOP in different macrophage subsets during liver fibrosis may advance our understanding of the involvement of CHOP in liver fibrosis.

#### Cardiac Fibrosis

It has also been suggested that CHOP may be involved in cardiac myocyte apoptosis, cardiac hypertrophy, and heart failure (135, 136). A high-fat diet fed to metabolically healthy, obese minipigs activates oxidative stress and ER stress with increasing expression of CHOP in myocardial fibrosis within the minipigs (137). In mice subjected to a transverse aortic constriction operation, ablation of CHOP can attenuate cardiac hypertrophy, cardiac dysfunction, and fibrosis with less apoptotic cell death (138) and alleviate myocardial reperfusion injury *via* attenuated myocardial apoptosis and inflammation (139).

### Cancer

In states of uncontrolled proliferation and insufficient vascularization (e.g., in cancer), conditions of low nutrient supply, such as hypoxia and oxidative stress, may trigger ER stress and subsequent UPR activation that have been documented in various human cancers (140). Studies have shown evidence of CHOP activation in various types of cancer cells (125, 141). Thus, CHOP-induced apoptosis in ER stress has significant implications for cancers. Furthermore, *CHOP* mutations are found in some human tumors, although whether or not they contribute to tumorigenesis remains unknown (142). Accumulating data suggest that CHOP impinges upon several aspects of cancer including tumor formation as well as progression of tumors once formed. Nonetheless, how CHOP activation exerts tumor-supporting or tumor-suppressive roles remains to be elucidated.

#### The Role of CHOP in Carcinogenesis

Most of the evidence supports an anti-oncogenic function of CHOP-induced apoptosis in a stressful environment. Indeed, apoptosis is a critical mechanism for maintaining tissue homeostasis through selective elimination of cells once they are damaged, mutated, or pose a threat to the organism, such as precancerous cells. During stress conditions, the human hematopoietic stem cell (HSC) pool maintains integrity by elimination of individual HSCs through PERK–eIF2α–ATF4–CHOP–GADD34 signaling induced apoptosis, hence preventing persistent cloning of oncogenic mutations and decreasing the risk of leukemogenesis (73). CHOP induction triggers apoptosis of premalignant cells to prevent malignant progression in a mouse lung cancer model (143). Hepatocyte-specific *CHOP* ablation increased tumorigenesis in high fat diet-induced steatohepatitis and HCC. This effect indicates a tumor-suppressive role of CHOP, perhaps *via* apoptosis of initiated hepatocytes in preneoplastic lesions (144). However, CHOP tends to promote specific oncogenic processes, at least in one case; when fused with FUS/TLS or EWS protein by genomic rearrangement (145, 146). The FUS–CHOP oncoprotein has been newly proved to induce metastasis *via* transcriptional induction of tumor-associated proteases, both in liposarcoma and fibrosarcoma cell lines, as well as an *in vivo* model (147). Specifically, DeZwaan-McCabe et al. (125) proposed that ISR-induced CHOP provokes inflammation and fibrosis followed by compensatory proliferation to promote chemical hepatocarcinogenesis (148). CHOP was upregulated both in genetic and DEN-induced mouse models of HCC, as well as human HCC. *CHOP^−/−^* mice were protected from DEN-induced oncogenesis in liver, which was also proven by Scaiewicz et al. (149). The latter further found a marked reduction of IFNγ levels and macrophages in CHOP-knockout tumors and ATF6 activation upstream of CHOP, implying that CHOP induction regulates inflammation and macrophage infiltration to promote hepatocarcinogenesis after DEN treatment. In actuality, the carcinogen DEN evokes DNA damage and the apoptosis induced by this damage partly contributes to chronic inflammation and release of tumor-promoting cytokines. Collectively, CHOP may play an anti-oncogenic role in the precancerous cells and an oncogenic role when expressed in macrophages.

#### CHOP in Cancer Development and Progression

During the progression of tumors, CHOP triggers the death of a number of tumor cells, which has been reviewed (3). In the case of hepatoma cells, CHOP mediates the autophagic apoptosis induced by apoptosis-stimulating protein of p53-2 (150). However, the tumor-supporting functions of CHOP have been newly indicated in certain cells. Thevenot et al. found that the MDSC with immunosuppressive activity within tumors aberrantly expressed CHOP without completely undergoing apoptosis. *CHOP* deficiency induced antitumor effects in a MDSC-dependent manner, suggesting an important role of CHOP in tumor tolerance and potential benefits of its inhibition for tumor immunotherapy (88, 151). Moreover, SPOP mutations fail to mediate CHOP degradation and suppress CHOP-induced apoptosis, which indicates CHOP involvement in the progression of prostate cancer is associated with SPOP mutations (9). A common hallmark of a tumor microenvironment is hypoxia, during which cancer cells can activate pathways to develop and progress, such as immune responses. CHOP expression, in the context of ER stress and TLR agonists, increases dendritic cell expression of IL-23 (152), which supports T helper 17 cell propagation and its function to promote immune response and tumor growth (153). Induction of autophagy by ATF4 and CHOP helps several human cancer cell lines adapt to hypoxia (96), whereas a previous study suggests that cyclophilin B mediates the adaptation of tumor cells to hypoxia through ubiquitin-dependent degradation of CHOP (154). Overall, further understanding of the pro- and anti-oncogenic roles of CHOP and information on the cell types where CHOP is activated or suppressed in different stages of cancer may provide insight into different carcinogenesis modalities and promote its implication for cancer therapy.

#### CHOP in Cancer Therapy

Here, we also highlight recent advances in therapeutic strategies for cancer treatment that involve CHOP. CHOP-induced cell death has been widely suggested as one of the strategies to ameliorate cancer (Table 1). First, experiments with cancer cells have demonstrated that CHOP-mediated DR5 expression is responsible for the caspase8-mediated apoptotic pathway (70). Therefore, various natural and synthetic products that enhance CHOP–DR5 signaling have been presented for treatment of a series of cancers (45, 71). Second, a selective CHOP inducer, sulfonamidebenzamide, was identified with pro-apoptotic and antiproliferative effects in multiple cancer cell lines (155). Asparagine was found as a CHOP inhibitor with anti-apoptotic function, and suppression of asparagine synthetase may restore CHOP-induced cell death and exert therapeutic benefit in solid tumors (156). Finally, CHOP forms a complex with C/EBPβ and decreases C/EBPβ-dependent ALDH1A3 expression in chemoresistant cell subpopulations. This mechanism may be responsible for butein-induced enhancement of chemoresistant cell apoptosis (157) and contributes to the treatment of non-small cell lung cancer with garcinol (158). The suppression of STAT3–NFκB activity by butein is a prerequisite for high levels of CHOP expression (157). Moreover, CHOP downregulated the anti-apoptotic p21 in cancer cells treated with TUN, thus enhancing chemotherapeutic drug efficacy (86). During radiotherapy with high-LET carbon ions, chloroquine co-treatment enhances apoptosis *via* IRE1–CHOP signaling *in vitro* and *in vivo* (159). Besides apoptosis, CHOP regulated androgen receptor degradation in prostate cancer cells treated with rosemary extract (160). All types of oncogenic FUS–CHOP fusion proteins can be inactivated by trabectedin through blockage of their binding to target promoters, both in a mice xenograft model and human cell lines, thereby exerting a selective antitumor activity (161).

**Table 1:** Strategies to target C/EBP homologous protein (CHOP)-mediated cell death for cancer treatment in preclinical models.

Cancer type	Treatment agents	Involved mechanisms and phenotype	Research models	Reference
Pancreatic cancer	CGK733	Induces calcium sequestration in reversible vesicles through PRKR-like ER kinase (PERK)-CHOP signaling and subsequent non-apoptotic/necrotic cell death	Cells	(207)

Hepatocellular carcinomas	IMB-6G	Induces mitochondrial-dependent apoptosis *via* PERK–CHOP signaling Induces IRE1α–ASK1–JNK mediated apoptosis	Cells	(208)
	Piperlongumine	Increases reactive oxygen species (ROS) and activates endoplasmic reticulum (ER)–mitogen-activated protein kinases (MAPKs)–CHOP signaling *in vivo* and *in vitro* to trigger cell death Suppresses migration/invasion	Cells, mice (xenografts)	(43)

Triple-negative breast cancer	YM155 (surviving suppressant)	Upregulates p38 mitogen-activation protein kinase (p38 MAPK)- and CHOP-mediated DR5 expression to induce apoptotic response Impairs cell growth and increases cytotoxic effect	Cells, mice	(209)

Non-small cell lung cancer	Obovatol	Activates CHOP-induced apoptosis	Cells	(210)
	Licochalcone A	Induces CHOP-dependent apoptosis and autophagy	Cells	(94)

Ovarian carcinoma	Tanshinone IIA	Activates extrinsic apoptosis by JNK–CHOP–DR5 signaling	Cells	(44)

Colon carcinoma	Apigenin	Activates CHOP-mediated intrinsic and extrinsic apoptotic pathways with ROS generation and Ca^2+^ release Exerts anti-proliferation and cell cycle arrest role	Cells	(211)

Colorectal cancer	Rapalogs and ATP-competitive mTOR inhibitors	Activates CHOP–DR5 axis-dependent extrinsic apoptosis pathway	Cells	(212)

Multiple myeloma	Histone deacetylase 4 inhibitor	Activates activating transcription factor (ATF)4–CHOP-induced apoptosis Enhances the cytotoxicity of ER stressor	Cells, mice	(213)

T-cell lymphoblastic lymphoma and T-cell acute lymphoblastic leukemia	LAT1 selective inhibitor	Induces ATF6, ATF4, elF2α, growth arrest and DNA-damage-inducible protein 34, p38 MAPK expression and triggers CHOP-dependent apoptosis Decreases activation of Akt and mTORC1 Decreases cell viability and proliferation	Cells, mice (xenografts)	(214)

T-cell acute lymphoblastic leukemia	Inhibitor of CK2α	Activates apoptosis induced by IRE1α and CHOP Downregulates PI3K/Akt/mTOR signaling and the levels of GRP78 Exerts cytotoxic and cytostatic effects	Cells	(215)

Oral squamous cell carcinoma	Celastrol	Induces cell death through PERK–eIF2–ATF4–CHOP signaling	Cells, murine embryonic fibroblasts	(216)

Human esophageal cancer	Neddylation inhibitors (MLN4924)	Induces ATF4–CHOP–DR5-mediated extrinsic apoptosis Triggers ATF4–Noxa axis-mediated intrinsic apoptosis	Cells, murine	(72)

Glioblastoma multiform	Isochaihulactone	Induces CHOP–NAG1-mediated apoptosis independent of PERK	Cells, mice (xenografts)	(217)

### Diabetes

In diabetes mellitus, the glucostatic cycle to maintain normoglycemia is dysregulated due to an insufficient mass of functioning pancreatic β-cells to synthesize the needed amounts of insulin for metabolism (162). For type 2 diabetes (T2D), insulin resistance under stress of excess nutrients, including hyperglycemia and hyperlipemia, causes progressive β-cell failure (163). Dissimilarly, in type 1 diabetes (T1D), β-cells are attacked by autoimmune activity and the workload of the remaining β-cells increases. Collectively, high demand of insulin synthesis and secretion overwhelms the capacity of β-cell ER and thereby activates UPR to compensate. As the process continues, terminal UPR leads to apoptosis of β-cells and the onset of diabetes (164).

Apoptosis has been the main focus of studies on β-cell dysfunction during diabetes, among which CHOP-induced apoptosis is the most studied, as it is a key event in the pathogenesis of diabetes (165). Indeed, numerous studies have found that islet cells from mice and patients with T1D or T2D manifests elevated levels of CHOP (7, 166). In 2002, studies conducted with Akita mice proved that genetic removal of CHOP alleviated β-cell loss and hereditary diabetes, which vividly links CHOP to β-cell apoptosis for the first time (167). *CHOP* deficiency also prevents oxidative damage with reduced ROS and thereby improves ER function in β-cells (168), while oxidative stress is proven as an important factor that gives rise to β-cell dysfunction in diabetes (169). Thus, past studies have identified drugs, such as vildagliptin (170), that promote β-cell survival by decreasing CHOP expression in diabetic mouse models, along with downregulation of ATF4 and TRIB3 in T2D db/db mice (171).

For T2D, it has been evidenced that CHOP is responsible for β-cell apoptosis and dysfunction both in genetic and diet-induced mouse models of T2D, as well as *in vitro* (168, 172). The PERK/eIF2α/CHOP signaling mediated β-cell sensitization to lipotoxicity and apoptosis under the challenge of palmitate (173, 174) and guanabenz (175). Ubiquitination and degradation of CHOP by cellular inhibition of apoptosis protein-1 prevented palmitate-induced lipotoxicity (173). Consistent with this notion, the inhibition effect of CHOP on adipocyte differentiation interferes with effective fatty acid storage, which may cause lipotoxicity. Furthermore, the human islet amyloid polypeptide (h-IAPP), also characteristic of T2D, induced dysfunction of autophagy and apoptosis through CHOP, but inhibition of CHOP alone may not be a durable therapeutic strategy for the β-cell toxicity of h-IAPP, considering multiple stress pathways are activated during this process (176). To unveil the specific mechanisms underlying β-cell apoptosis in T2D, researchers found that CHOP regulation of puma is essential for the apoptotic pathway during glucotoxicity T2D (177). Another critical event is that CHOP downregulates p21 to trigger β-cell apoptosis due to glucotoxicity, thus promoting the onset of T2D (85, 178). As such, the chemicals that inhibit CHOP expression protect β cells from apoptosis and dysfunction, such as 1,2,3-triazole derivatives (179).

For insulinopenic T1D, autoimmunity triggers an inflammatory response along with cytokine release which induces ER stress in β-cells. CHOP contributes to cytokine-induced apoptosis of β-cells *via* mitochondrial apoptotic pathways and indirect pro-inflammatory responses, indicating the role of CHOP in T1D. Mechanistic studies have shown that CHOP knockdown in insulinoma cell lines protected against the downregulation of anti-apoptotic BCL-2-like proteins, Bcl-2 and Mcl-1, while decreasing NF-κB activity and expression of its target genes, including inducible NO synthase (iNOS) and TNF receptor superfamily member 6 (FAS) (180). CHOP blocking by siRNA partially protected human beta cells against cytokine-induced apoptosis independent of NO, whereas CHOP induction was NO dependent and could be inhibited by iNOS blocker in rat insulin-producing cells (181). Moreover, CHOP acted as a mediator of β-cell apoptosis in islets deficient for Gata4, which belongs to a group of β-cell survival factors that contribute to T1D risk when they undergo mutations (182).

Overall, cumulating evidence suggests that CHOP is involved in the pathogenesis of diabetes, predominantly T2D, in response to glucotoxicity, lipotoxicity, as well as oxidative stress and islet amyloid derived from IAPP. With regard to diabetic complications, there is also some relevance to CHOP. For example, during murine DN, ATF6-dependent CHOP activation was induced by defective insulin signaling due to impaired nuclear translocation of sXBP1 in podocytes (34), whereas *CHOP*-null mice gained protection from DN (183). Tubules of diabetic mice and patients showed increased levels of CHOP protein, and other than apoptosis, CHOP-induced expression of fibronectin in tubule cells (123). As for diabetic cardiovascular complications, including diabetic cardiomyopathy, IL-1β-induced myocyte apoptosis was mediated by the IRAK-2/CHOP pathway (184). CHOP-induced apoptosis was partly targeted by Ginsenoside Rg1 to ameliorate diabetic myocardial damage (185). Correspondingly, myocyte apoptosis and cardiac dysfunction induced by methylglyoxal were attenuated in *CHOP*-null mice (62). Furthermore, streptozotocin-induced diabetic *CHOP^−/−^* mice manifested not only reduced hyperglycemia but also lessened severity of oxidative-nitrative stress in their sciatic nerve and in their eventual diabetic peripheral neuropathy (186).

### Neurodegeneration

Neurodegenerative diseases are hallmarked by progressive loss of neuronal function. Many risk factors including aging, oxidative stress, and gene mutations of neurodegenerative process can cause toxic accumulation of misfolded proteins, which ultimately leads to neuronal cells undergoing ER stress-induced apoptosis. As a key player in ER stress and oxidative stress, CHOP expression is found to be elevated in many disorders related to neurodegeneration, such as the Parkinson disease (PD) (187), and CHOP induces neuronal apoptosis, which has been proposed as a target of treatments for some neurodegenerative diseases (188). In some cases, disruption of CHOP exerts a neuroprotective role through yet unknown mechanisms (189). We list here important studies on CHOP involvement in neurodegenerative diseases in recent years.

A prominent clinical hallmark of Alzheimer’s disease (AD) is progressive cognitive impairment. AD is attributed to pathological deposits of neurofibrillary tangles formed by hyperphosphorylated tau aggregates and abnormal aggregation of amyloid-β (Aβ) plaques. In the AD brain, ATF4 synthesis in axons locally exposed to Aβ_1–42_ triggered retrograde cell loss through CHOP, and conversely, *CHOP* deletion hindered Aβ_1–42_-mediated neurodegeneration (190). In the mouse model of AD and neuroblastoma cells, researchers analyzed the effect of palmitate and noticed that CHOP indispensably mediated increased β-site APP-cleaving enzyme 1 (BACE1) activity and ensuing Aβ production, but only partially (191). In agreement with this, silencing *CHOP* expression attenuated NF-κB activation and its binding to the BACE1 promoter, thus reducing Aβ production induced by 27-hydroxycholesterol (192). CHOP knockdown also alleviated the negative regulation of C/EBPα binding to the leptin promoter and subsequent leptin expression, which is able to decrease Aβ genesis and tau phosphorylation (193). As for PD, CHOP and ATF4 play a key role in regulating Trib3 and apoptosis in cellular models of PD, as evidenced by the protective role of CHOP and ATF4 knockdown in 6-OHDA and MPP(+) models (194). Inhibition of CHOP-mediated crocin-induced neuroprotection in the PD model through Wnt pathway *in vitro* (195).

In S63-deletion mice of Charcot–Marie–Tooth (CMT) disease type 1B, *CHOP* deletion decreased demyelination and rescued their motor deficit (196). Mechanistic studies subsequently revealed that CHOP targeted GADD34 to reactivate translation in the nerves of this model (197). Surprisingly, CHOP ablation did not rescue the abnormalities of Schwann cell development in R98C mouse model of type 1B CMT (198). Prion-related diseases are another type of neurodegenerative disorder, and accumulation of prion protein (PrP) defines the pathobiology. Upregulation of CHOP through the PERK pathway is a pathogenic factor of neurodegeneration induced by the membrane-tethered flexible tail of PrP (199). A role of CHOP in mild spinal cord injury is indicated by enhanced neuronal functional recovery in CHOP-deficient mice, partly due to decreased oligodendrocyte apoptosis (200). Moreover, CHOP and caspase12 induced neuron apoptosis at later stages of chemical hypoxia (201). In the context of neurodegeneration in retinas, a sustained upregulation of CHOP can result from optic nerve injury and *CHOP* deficiency increased the survival of retinal ganglion cells (202). Apoptotic cell death of photoreceptors was also mediated by CHOP in retinas deficient in autophagosomes (203). Likewise, in brain astrocytes, MA mediated CHOP upregulation downstream of the activation of all three ER stress pathways, which together lead to apoptosis *via* intrinsic caspase cascade (204). This arises as the mechanism of MA-mediated neurodegenerative effects. The PERK–eIF2α–ATF4–CHOP pathway mediated sevoflurane-induced neuroapoptosis in neonatal brains (205). Moreover, CHOP expression in the brain plays a pivotal role in the negative regulation of two neurotrophic cytokines, leptin and insulin-like growth factor-1 by palmitate (206).

## Summary and Perspectives

In summary, induction of CHOP is converged from the regulation of UPR, ISR, and MAPKs signaling in response to various cellular stress conditions, including ER stress and ROS. CHOP can be protective for cell survival *via* regulating autophagy in early stages (before irreversible ER stress). This stress-responsive transcription factor has been extensively recognized as the link between prolonged protein-folding stress, namely ER stress and apoptosis. To the best of our knowledge, no studies have yet shown that CHOP directly leads to apoptosis. CHOP indirectly regulates apoptosis by controlling the expression of pro-apoptotic or anti-apoptotic genes. Therefore, we have constructed a signal network depicting canonical and emerging targets of the CHOP-dependent apoptotic pathway, including the BCL-2-mediated intrinsic and DR5-mediated extrinsic apoptotic pathway. Collectively, they can result in protein aggregation, disturbance of redox status, and mitochondrial function to culminate in apoptosis. Inhibition of CHOP is an approach to improve the survival and function of cells. However, in certain conditions, CHOP expression does not induce cell apoptosis. Given the dual role of CHOP, whether it is more a pro-apoptotic or a protective factor remains to be defined in specific cell types. Cells behave differently to CHOP induction owing to the intensity and duration of stress, as well as distinct cell and disease context.

C/EBP homologous protein impinges upon different process such as autophagy, apoptosis, and cell differentiation. From an overview of its impact on different diseases so far, apoptosis is a major cellular function of CHOP that involves it in pathological processes for a wide range of diseases. CHOP-dependent apoptosis may exert amelioration or aggravation effects on different diseases. It has been increasingly implicated as a treatment strategy in the context of cancer and more *in vivo* research is needed to evaluate the efficacy. In addition, the emerging roles of CHOP in the progress of fibrosis and regulation of macrophage polarization open up new avenues for future research. The broader functions and molecular mechanisms of CHOP in physiopathology will continue to be unveiled to target it in potential therapeutic strategies.

## Author Contributions

Each author has participated sufficiently in the work to take public responsibility for appropriate portions of the content.

## Conflict of Interest Statement

We declare that none of the authors have any financial and personal relationships with other people or a third party that can inappropriately influence the quality of the work presented in this manuscript. There is no professional or other personal interest of any nature or kind in any product, service, and/or company that could be construed as influencing the position presented in, or the review of, the manuscript entitled, “Transcription Factor C/EBP Homologous Protein (CHOP) in Diseases.”
